# Supplementary figures and images for: Dengue NS1 interaction with lipids alters its pathogenic effects on monocyte derived macrophages
Source: J Biomed Sci. 2024 Sep 4;31:86. doi: 10.1186/s12929-024-01077-8 (PMC11373103; doi:10.1186/s12929-024-01077-8)

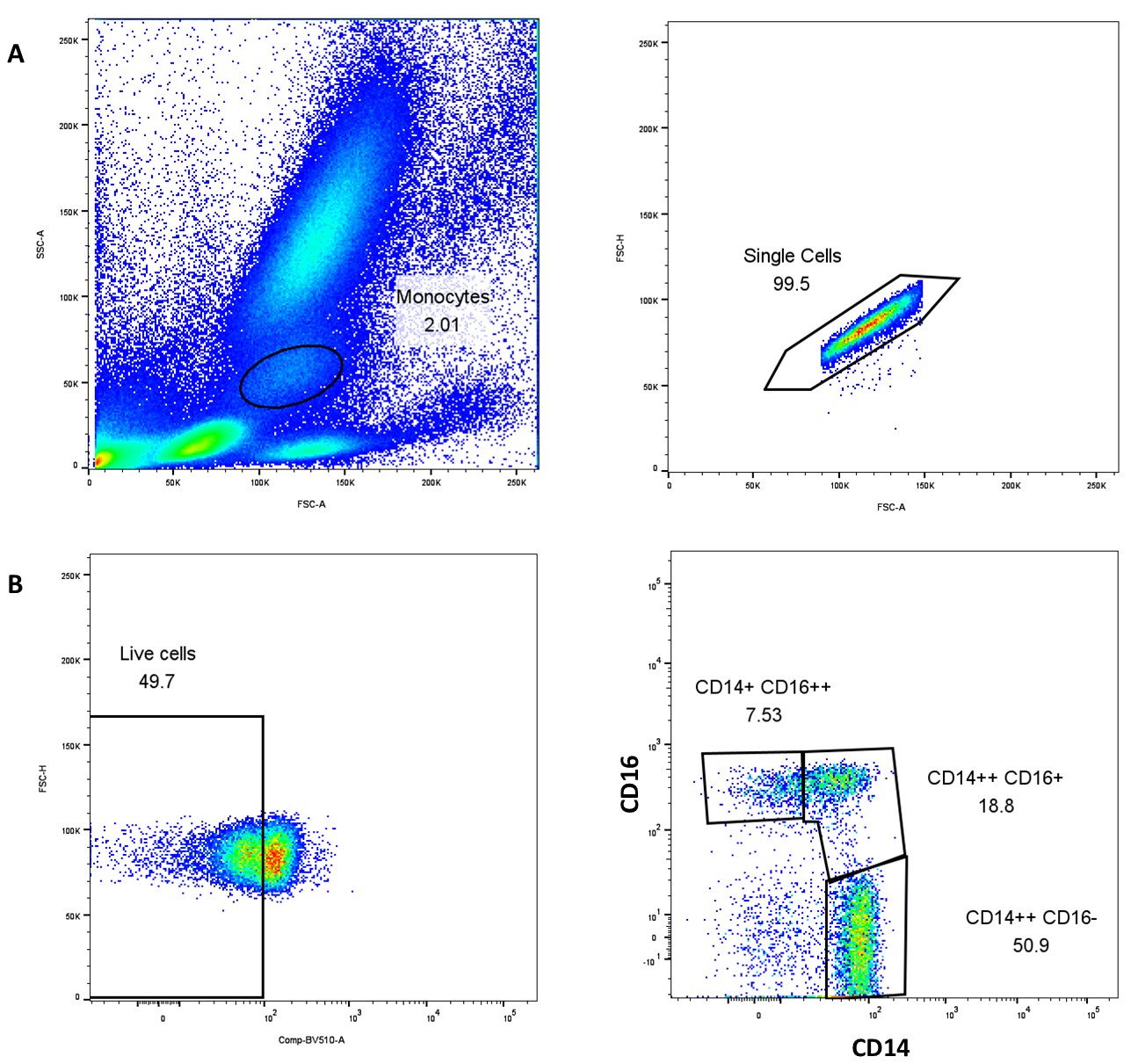

Supplement: Supplementary file 1 — Fig. S1. Monocyte phenotype example results of an individual. (A) Singlets were selected from the population of gated monocytes. (B) Monocyte subsets were selected from the live cell population and CD14 high CD16 low , CD14 high CD16 high , CD14 dim CD16 low and CD14 dim CD16 high phenotype percentages were obtained. [file 12929_2024_1077_MOESM1_ESM.jpg]
