# Supplementary material for: Dengue NS1 interaction with lipids alters its pathogenic effects on monocyte derived macrophages
Source: J Biomed Sci. 2024 Sep 4;31:86. doi: 10.1186/s12929-024-01077-8 (PMC11373103; doi:10.1186/s12929-024-01077-8)
Supplement: Supplementary file 2 — Table S1. Past dengue disease severity details and the year of experiencing dengue. [file 12929_2024_1077_MOESM2_ESM.docx]

**Supplementary Table 1:** Past dengue disease severity details and the year of experiencing dengue

| Category | Sample ID | Sex | Age | Year of infection |
| --- | --- | --- | --- | --- |
| DF | 20 | F | 28 | asymptomatic |
|  | 21 | F | 26 | 2017 (OPD) |
|  | 22 | M | 29 | 2017, 2019 |
|  | 23 | M | 43 | 2017 |
|  | 24 | M | 33 | 2019 |
|  | 25 | F | 50 | asymptomatic |
|  | 26 | F | 33 | asymptomatic |
|  | 27 | F | 27 | asymptomatic |
| DHF | 30 | F | 26 | 2005 |
|  | 31 | M | 29 | 2013, 2021 |
|  | 32 | F | 33 | 2017 |
|  | 33 | M | 28 | 2000 |
|  | 34 | F | 37 | 2022 |
|  | 35 | F | 30 | 2019 |
|  | 36 | F | 23 | 2023 |
|  | 37 | M | 26 | 2007 |
