# Supplementary material for: Dengue NS1 interaction with lipids alters its pathogenic effects on monocyte derived macrophages
Source: J Biomed Sci. 2024 Sep 4;31:86. doi: 10.1186/s12929-024-01077-8 (PMC11373103; doi:10.1186/s12929-024-01077-8)

**Supplementary figure for Reviewer #4 comments Q14:**

Morphological Changes in Monocytes Seeded in 96-well cell culture plates over the experiment. (A). On the day of monocyte separation, cells were seeded 1.25 x10^5^ cells per well(d0) (B). 7 days post incubation to convert them into MDMs (d7). All wells displayed uniform cell density and morphology, with no discrepancies observed across the samples.

On d7, MDMs were treated with NS1 and lipid mediators. The following day (d8), supernatants were harvested for IL-1β ELISA and cells were processed for RNA extraction. Photographs of the cells were taken on d8 prior to the removal of the supernatants.

(C). Cells treated only with NS1 (D). Negative control (media only)

(E.) Cells treated only with LPS (I) Cells treated with NS1 and LPS

(F) Cells treated only with PAF (J) Cells treated with NS1 and PAF

(G) Cells treated only with HDL (K) Cells treated with NS1 and HDL

(H) Cells treated only with LDL (L) Cells treated with NS1 and LDL


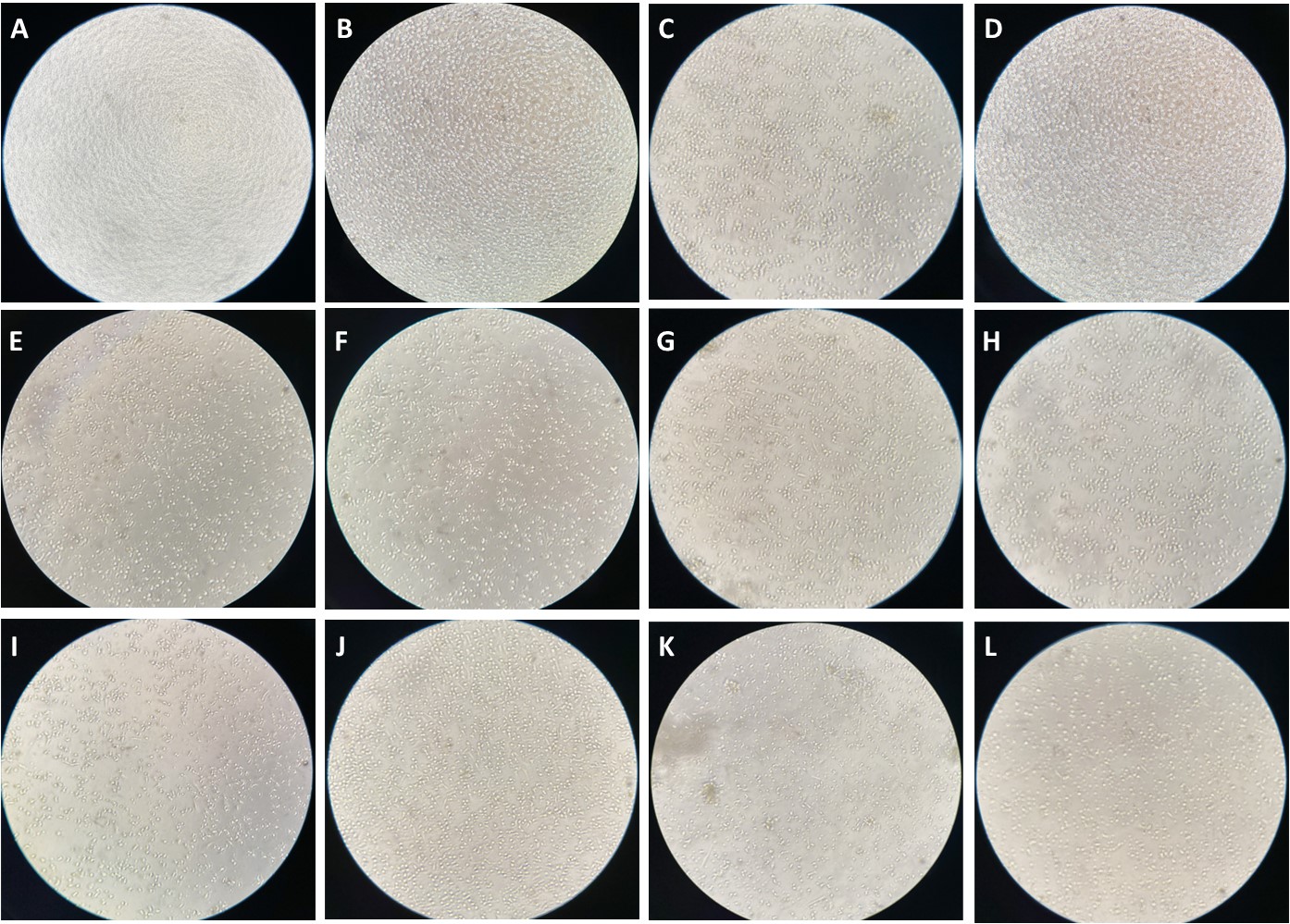

Supplement: Supplementary file 3 — Table S2. Past dengue disease severity details and the year of experiencing dengue. [file 12929_2024_1077_MOESM3_ESM.docx]
